# Mutations in circulating mitochondrial DNA: Cassandra of oral cancer?

**DOI:** 10.18632/oncotarget.567

**Published:** 2012-07-24

**Authors:** Eugene S. Kandel

**Affiliations:** Roswell Park Cancer Institute, Department of Cell Stress Biology, Elm & Carlton St., Buffalo, NY

The ability to predict whether a cancer will recur at either the primary or a secondary site after a therapeutic intervention is of major importance in clinical oncology. Despite tremendous progress in this field, direct detection of micrometastasis remains a daunting and uncertain task, especially since frequent biopsies are associated with considerable morbidity and health care cost. Also, existing limits on direct detection of cancer cells complicate determination of safe excision margins, which should be minimally sufficient to prevent local recurrence. This is especially challenging for such cancers as lentigo maligna melanoma, which may present with sparse malignant cells scattered along a large area of non-malignant tissue. A similar challenge is faced when managing conditions like Barrett's esophagus, which have a modest chance of malignant progression anywhere within a large area of metaplasia.

In part, the likelihood of a primary tumor giving rise to metastases could be inferred from the molecular signature of the tumor itself. However, a high-risk mutation may escape detection because it is present in only a minute fraction of the tumor or has happen at a metastatic site. Also, standard histopathological analysis of smaller tumors often leaves little material for molecular testing. Furthermore, samples of a primary tumor may not be available when a patient is monitored for disease recurrence later in life.

In this regard, patient's bodily fluids, and blood in particular, offer a desirable source of biomarkers: those fluids could be safely repeatedly collected and may reflect contribution of cells of different types and physical locales. In fact, several blood-based tests, such as the one for prostate-specific antigen, are mainstream tools of clinical oncology. The challenge is to identify a molecular signature specific enough to tell cancer recurrence from the lack of the disease and from non-malignant conditions, and yet applicable to most cases of at least one cancer type. In this context, nucleic acids offer a benefit of an easily amplifiable signal and tunable selectivity based on sequence-specific recognition[1]. Wide acceptance of the role of microRNAs in oncogenesis [2, 3] prompted their evaluation as prognostic and diagnostic markers. Interestingly, despite very promising development in that area, the magnitude of the change in miRNA abundance is quite modest and the actual origin of the biomarker miRNAs is controversial (e.g. [4]).

The question about the origin also applies to the studies of circulating cell-free DNA. The amount of circulating genomic DNA is affected by a variety of pathological conditions, but there are avenues to a more informative assay. First, a test could selectively detect pre-defined oncogenic mutations. Such mutations are unlikely to come from normal cells, but they may be too specific for cancers where the same oncogenic phenotype may arise from a variety of equivalent but distinct mutations. Second, there are emerging technologies to detect changes in the methylation status of specific regions of genomic DNA[5]. Such changes may either drive oncogenesis or indicate other perturbations in signaling events. In the latter case, such markers may be preferred over the mutational ones: equivalent signaling alterations may result from any of many individual mutations, and the same assay may apply to a larger subset of a given cancer type. As in the case of miRNA, the source of the informative methylated DNA does not have to be the tumor per se, but may be its stroma or the immune system. Encouragingly, methylation patterns may discriminate between cancerous and non-cancerous diseases of the same organ[5].

The tests of either mutations or methylation of cell-free circulating genomic DNA face another challenge: generally, only one or two marker molecules are expected to be released per cell. However, one notable exception is mitochondrial DNA (mtDNA). Mitochondrial genome is present at a high copy number per cell and undergoes frequent alteration in cancers [6, 7], thus being an attractive source for biomarker development. The feasibility of this is suggested in the current issue of *Oncotarget* by Uzawa and colleagues, who have found common mtDNA alterations in human oral cancer and have detected these mutations in the patients' serum. A combination of quantitative PCR with high-resolution melting curve analysis of three different mtDNA regions revealed that the patients whose tumors would recur had significantly higher levels of mutant mtDNA in their serum four weeks post surgery. The sensitivity and specificity of mtDNA-based tests exceeded 80% and 90% respectively. Mutant mtDNA was detectable in postoperative serum in only 3 out of 45 non-recurring cases, and even in those it declined, while in all the 16 recurrent cases it persisted or increased. Thus, the test could be useful for the patients for whom pre- and postoperative samples could be compared and also for those for whom no prior samples exist. The test measured the fraction of mutant out of total recovered mtDNA, and it is intriguing whether it could be improved by using “two-spin” plasma [8] instead of serum (e.g by reducing cellular contamination). Of course, an extensive prospective study is needed before the utility of this approach is established. Thorough controls for possible contamination during sample preparation and PCR would be central to such a study. Another issue in whether the phenomenon is unique to oral cancer, since similar attempts in other malignancies did not achieve equally impressive classification of clinical cases (reviewed in [1]), and whether it could be applicable to other conditions (e.g. aging) where mitochondrial dysfunction is common [9]. Indeed, the research on circulating mtDNA spans over a decade, yet it failed to attract as much attention as that of other circulating biomarkers. While the prior skepticism about the predictive value of mutant mtDNA might have been warranted, the report by Uzawa et al. suggests that this biomarker should not follow the fate of the Trojan prophetess whose potentially life-saving insights remained unheeded.
